# Experimental Investigation and Numerical Analysis Regarding the Influence of Cutting Parameters on the Asphalt Milling Process

**DOI:** 10.3390/ma17143475

**Published:** 2024-07-13

**Authors:** Marius Gabriel Petrescu, Teodor Dumitru, Eugen Laudacescu, Maria Tănase

**Affiliations:** Mechanical Engineering Department, Petroleum-Gas University of Ploiești, 100680 Ploiesti, Romania; pmarius@upg-ploiesti.ro (M.G.P.); leugen@upg-ploiesti.ro (E.L.)

**Keywords:** milling parameters, experimental stand, DEM, asphalt concrete, cutting forces, DOE

## Abstract

Abrasion wear is a significant concern for cutting tools, particularly when milling asphalt concrete due to the presence of hard mineral aggregate particles. The pressure exerted on the cutting tool by the chipped material and the resulting cutting forces directly influence tool wear. To estimate the cutting forces in asphalt milling, the authors propose using either laboratory experiments or cost-effective Discrete Element Method (DEM) modeling—by simulating the real conditions—as direct measurement under real conditions is challenging. This article presents results from an original experimental program aimed at determining the cutting forces during asphalt pavement milling. A specialized stand equipped with a moving plate and recording devices was designed to vary milling depth, rotational speed, and advance speed. The experimental results for horizontal force values were compared with numerical results from DEM modeling. It was found that both increasing the milling depth and the advance speed lead to higher cutting forces. Generally, DEM modeling trends align with experimental results, although DEM values are generally higher. The statistical analysis allowed identification of the milling depth as the most significant parameter influencing cutting force and the optimal combination of milling parameters to achieve minimum horizontal force acting on cutting tooth, namely, 15 mm milling depth and 190 mm/min advanced speed.

## 1. Introduction

**Figure 1:**
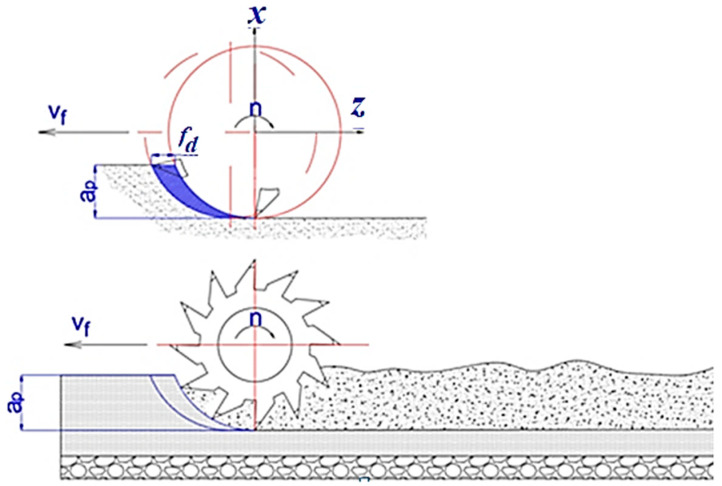
Milling schematization: *n*—milling drum rotation speed, rpm; *v_f_*—speed in the advanced direction, m/min; *a_p_*—milling depth, mm; *f_d_*—tooth advance, mm/tooth [3].

Asphalt concrete, widely used in road construction due to its durability and cost-effectiveness, undergoes milling processes to maintain or rehabilitate road surfaces. Understanding the dynamics of these milling processes is essential for optimizing efficiency, extending equipment lifespan, and ensuring the quality of road surfaces. Central to this understanding is the analysis of horizontal resistance forces encountered during asphalt milling, which are influenced by various cutting parameters (Figure 1) such as milling depth, rotation speed, and advanced speed [1,2,3,4,5,6,7,8]. Accurately estimating the cutting force is crucial in designing cutting tools for milling process [9,10,11,12,13].

Developing new machinery and enhancing the performance of current ones necessitate addressing numerical modeling of the milling process through a robust mathematical model and developing software that is capable of calculating the working tool parameters. Achieving this objective involves accurately simulating the milling process. Methodologically, experimental studies examining the cutting processes of diverse road construction materials prove valuable, as they yield highly dependable results that are applicable in practice.

To date, various researchers have undertaken multiple endeavors to investigate the asphalt milling process and evaluate the forces acting on the cutting tools through experimental [4,5,6,14,15,16,17,18] and numerical [1,3,7,8,19,20,21,22,23,24] methodologies.

The numerical DEM determination of the cutting resistance forces developed on the cutting tooth during the asphalt milling process is reflected in the works [1,3], where the authors investigated the influence of the milling parameters (milling depth, advanced speed, drum rotation speed, angle of attack).

Other conducted studies [5] enabled the determination of the correlation between cutting strength and the percentage of wear of the cutting element, as well as the thickness of the chip being cut, analyzing the impact of the carbide tip on the cutting element of a milling machine during asphalt concrete milling.

Similarly, article [4] focuses on the findings of a study regarding the cutting resistance of road asphalt concrete during the removal of worn coatings by milling machine working bodies. The experimental work involved determining both the horizontal and vertical components of the resistance force during the cutting of asphalt concrete. This research was conducted using a mechanical stand equipped with a movable plate and recording equipment. Four different grades of asphalt concrete were tested, with a two-factor experiment conducted for each material brand. The horizontal and vertical components of the cutting resistance forces were analyzed with respect to the chip cross-sectional area, asphalt concrete grade, and cutting element type. The collected data support the creation of a mathematical model for asphalt concrete milling. This model aids in determining loads on the working body and the process’s energy intensity. Moreover, it assists in establishing an optimal range of equipment operating modes and guiding mode selection according to the type of asphalt concrete.

The scientific work [18] details the development of a pendulum stand utilized for studying asphalt concrete milling processes that employs a single-tooth cutter, enabling the determination of cutting work, along with average and instantaneous cutting forces. Additionally, the study presents test results for cutting elements interacting with some asphalt concrete samples of various grades commonly used in road construction. Notably, the article highlights the non-linear relationship between cutting resistance forces and both temperature and penetration thickness of the cutting element. It is emphasized that operating with minimal tooth penetration reduces energy efficiency. Furthermore, the impact of temperature on cutting resistance is found to be less significant in asphalt concrete with higher gravel content. Another scientific work [13] investigates the total cutting force and geometric surface parameters in the end milling of NiTi alloy. It explores the effects of cutting speed (vc), feed per tooth (fz), and radial depth of cut (ae) using a Box–Behnken experimental design. Through response surface methodology (RSM), mathematical models were developed to predict cutting forces and 3D surface characteristics. These models elucidate the relationships between input parameters and outputs, facilitating the optimization of NiTi alloy milling.

Also, experimental studies were conducted in [17] to determine the wear resistance of the cutters and the load on the road milling cutter drive. Moreover, analytical investigations delve into the impact of tribological parameters on cutter wear intensity. By solving dynamic equations in a viscoelastic medium, the geometric shape of the cutter is optimized. Mathematical relationships are derived, revealing how the cutter’s geometric dimensions affect pressure and tool wear intensity.

Based on in-situ experiments, the goal of work [15] is to introduce a model for assessing the wear rate of picks on mobile asphalt milling machines during the cold milling of asphalt pavement. This model is then applied using field experimental data to evaluate the durability of picks from various manufacturers. The results reveal that the length of the pick, carbide tip diameter, and steel body diameter of picks from both manufacturers decrease proportionally with the milled asphalt pavement surface. Through Fisher’s criterion, it was established that the variances of the reduction in these geometrical parameters are equal and conform to a normal distribution according to Kolmogorov’s criterion. Moreover, all calculated statistics for Student’s criterion exceeded the critical values, indicating a significant difference in wear intensity between picks from the two manufacturers.

However, while the existing literature has extensively explored the theoretical and numerical aspects of asphalt milling, there remains a notable gap in experimental investigations. This study addresses this gap by presenting both the experimental measurements and numerical simulations of horizontal resistance forces during asphalt milling. By systematically varying cutting parameters like milling depth, rotation speed, and advance speed, this research aims to provide comprehensive data that validate numerical simulations and enhance the understanding of asphalt milling dynamics by simulating the real conditions.

The experimental aspect involved the design and implementation of a specialized stand equipped with recording devices to measure horizontal resistance forces during milling operations. Through the systematic variation of cutting parameters, including milling depth, rotation speed, and advance speed, comprehensive data were collected to assess their impact on the milling process.

**Figure 2:**
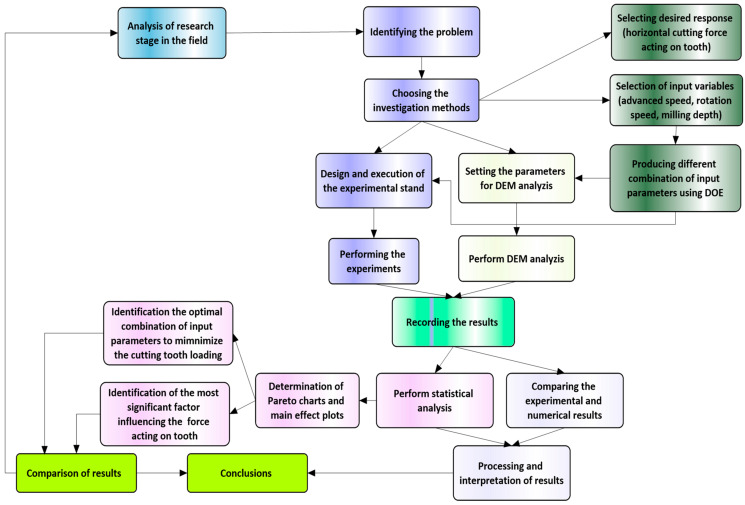
Flowchart of the steps in the present investigation.

In parallel, numerical simulations based on the Discrete Element Method (DEM) were conducted to complete the experimental findings. DEM offers a computational framework to model the complex interactions between milling tools and asphalt concrete, allowing for the prediction of horizontal resistance forces under different cutting conditions. By comparing experimental results with numerical simulations, this study aims to validate the efficacy of DEM in simulating the asphalt milling process and analyze its potential for further investigations (Figure 2 shows the steps in the present work).

The importance of this research rests in its capacity to enhance the understanding of asphalt milling dynamics and optimize milling operations. By identifying the influence of cutting parameters on horizontal resistance forces, road construction professionals can make informed decisions regarding equipment settings and operational strategies to improve efficiency and quality. This helps not only to identify the optimal milling regimes from the point of view of the forces developed, but also to keep the wear of the cutter teeth under control in order to increase their durability. Additionally, the validation of numerical simulations against experimental data contributes to advancing computational tools for modeling and analyzing asphalt milling processes.

## 2. Materials and Methods

### 2.1. Description of the Experimental Stand

**Figure 3:**
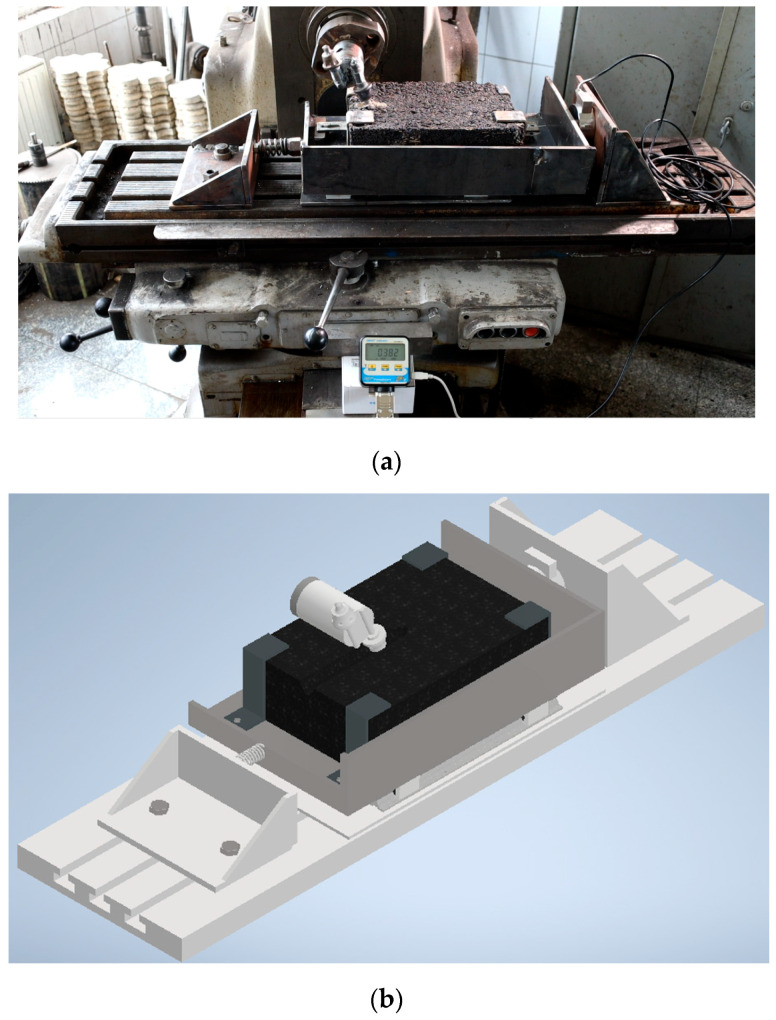

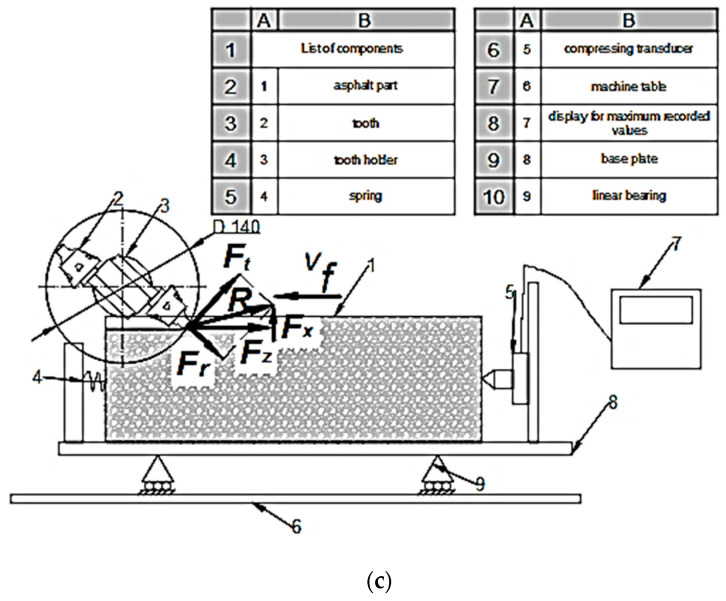
Milling machine FU 32 × 132, with the device for milling asphalt and measuring the horizontal component of the chipping force: (**a**) overall photo; (**b**) 3D drawing; (**c**) principle scheme.

In the scope of the experimental studies in laboratory conditions, the special stand presented in Figure 3 was designed, having the characteristics in Table 1. The stand reproduces the real conditions, from the point of view of the elements that participate in the milling process. During the construction of the stand, teeth taken from real milling cutters were used to make the milling cutter, and the processed part was represented by asphalt concrete blocks having the composition of the asphalt coating. The milling process was analyzed by investigating the interaction between a single cutting element (a single tooth of the milling cutter, knowing that, in practice, asphalt milling involves one or two teeth for each cross-section relative to the milling axis) of the milling drum and the asphalt sample. This approach facilitated the determination of the reaction forces during the cutting process, as well as the ability to adjust various geometric and dynamic parameters of the cutting processes over a wide spectrum, including milling depth, rotational speed, and the speed and direction feed rate of the displacement of the milling cutter. Asphalt concrete samples were used for the experiment. They were made of asphalt concrete having the same composition (Table 2) as the asphalt concrete used in the manufacture of roads (asphalt concrete samples were taken from a batch manufactured at the Strabenbau Logistic asphalt station). The asphalt concrete samples were cast and compacted in molds with dimensions of 350 × 240 × 100 mm. After formatting, the samples were kept in the ambient atmosphere for 240 days, in order to obtain a natural asphalt consistency (corresponding to road surfaces).

**Figure 4:**
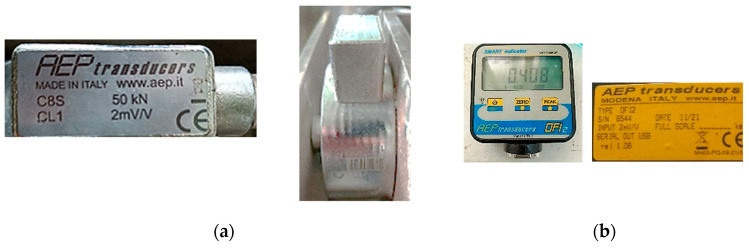
Devices used for recording forces values: (**a**) Compression transducer; (**b**) Recorder with display.

We utilised a compression transducer, type MR02, max. 50 kN, Accuracy ±0.5% of maximum value (Class 1) (Figure 4a). Visualization of the force values and recording of its maximum value were carried out with a recorder with display (Figure 4b).

**Figure 5:**
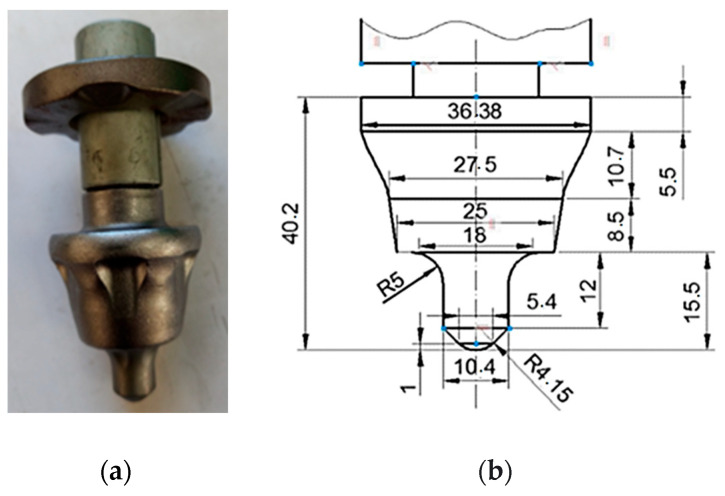
The geometry of milling cutter tooth: (**a**) real Wirtgen tooth; (**b**) dimensions (in mm) [3].

The cutter tooth used was Wirtgen type (as shown in Figure 5), having the main body made of steel and the tip from tungsten carbide.

The technological parameters, according to Figure 1, are presented in Table 3.

### 2.2. DEM Investigation

**Figure 6:**
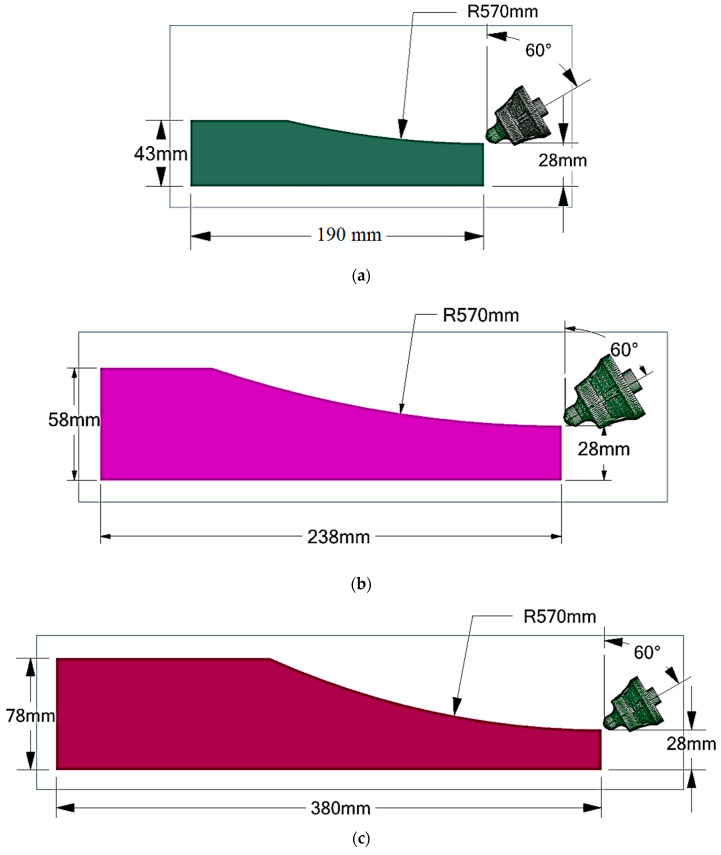
Space Claim model corresponding to different milling depths: (**a**) *a_p_* = 15 mm; (**b**) *a_p_* = 30 mm; (**c**) *a_p_* = 50 mm.

The numerical investigation performed was the Discrete Element Method (DEM) with Rocky 2022 R2 software. To enhance computational efficiency, spherical particles with a diameter of 8 mm were employed. All details regarding the setting for the numerical simulation can be found in our previous paper [3]. Geometrical models for DEM simulations of both tooth and asphalt were generated using the Space Claim module (as shown in Figure 6).

**Figure 7:**
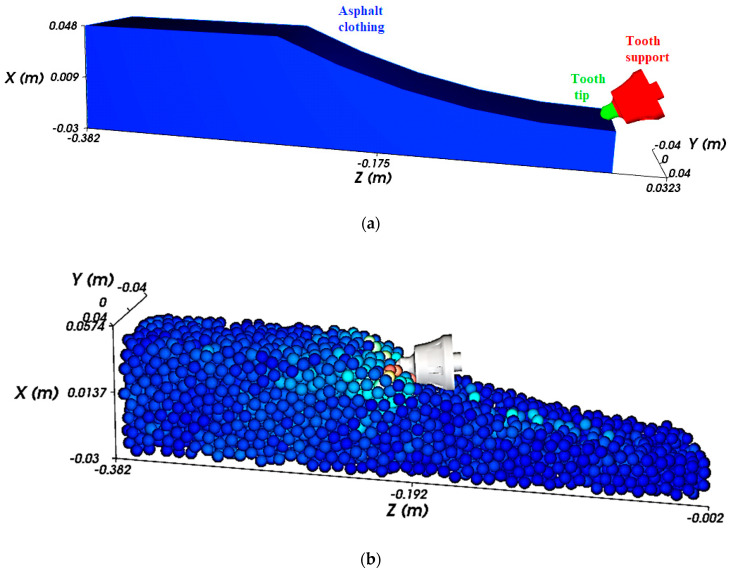
The model used for numerical analysis: (**a**) the geometrical model imported in Rocky; (**b**) DEM milling process simulation.

Different STL files with various depths were subsequently incorporated into the Rocky 2022 R2 version software to simulate different milling scenarios. Figure 7 illustrates the 3D cutting model of the asphalt.

### 2.3. Theoretical Aspects Regarding the Asphalt Milling Process

The milling of the asphalt pavement is carried out on the principle of milling in the opposite direction, according to the cutting scheme in Figure 3.

The average value of the tangential components, *F_t_*, is calculated with the following relation [25]:

*F_t_* = (*C* · *t^x^* · *f_d_^y^* · *a_p_^u^* · *z*) · *K*/(*D^q^* · *n*^w^) (1)

where *C* is milling force coefficient; *t* is the contact length of the tooth edge with the semi-finished product in mm; *f_d_* is the tooth advance in mm/tooth (for a single tooth acting in the plane of the cross-section through the asphalt cutter [3], *f_d_* = *f*); *a_p_* is the milling depth in mm; *z* is the number of cutter teeth in a cross-section (for asphalt cutters, in general, *z* = 1); *K* is a coefficient that depends on the used material; *D* is the cutter diameter in mm; *n* is the cutter rotation speed in rpm; and *x*, *y, u, q,* and *w* are coefficients.

The horizontal component of the milling force, *F_z_*, is determined as [25]:

*F_z_* = (1.1 … 1.2) · *F_t_* (2)

In the situation with the same material conditions, the same milling depth, the same feed rate and rotation speed, and the same number of cutter teeth, if different diameters *D*_1_ and *D*_2_ are used, the relationship between the forces in the horizontal direction (the cutter advance direction) is [25]:

*F_t_*_1_ · *D*_1_*^q^* = *F_t_*_2_ · *D*_2_*^q^* (3)

which, based on Relation (2), becomes:

*F_z_*_1_ · *D*_1_^q^ = *F_z_*_2_ · *D*_2_*^q^* (4)

For the case analyzed in this work, corresponding to the two approaches—the simulation of the milling process of the asphalt pavement using DEM and the experimental analysis, in the laboratory, of the interaction of the milling machine with the asphalt pavement—we considered the following:

*F_z_*_1_—horizontal component of the milling force, determined by DEM simulation;

*F_z_*_2_—horizontal component of the milling force, determined by laboratory experiment;

*D*_1_—milling diameter, *D*_1_ = 1140 mm (corresponding to milling machines ECO 2000 made by Benninghoven);

*D*_2_—diameter of the milling device (mill) of the experimental stand, *D*_2_ = 140 mm.

In these conditions, between the two studied cases, by referring to the forces in the horizontal direction, a relationship is established:

*F_z_*_2_ = *F_z_*_1_ · (*D*_1_/*D*_2_)*^q^* (5)

According to [26], the hardness of asphalt pavement is estimated at 1.3 Mohs. However, depending on the content and quality of mineral aggregates embedded in asphalt concrete, its hardness can increase considerably [27], reaching a Mohs value of 7, comparable to cast iron, an alloy that has a hardness between 3 and 7 on the Mohs scale, depending on the shape of the graphite and the iron matrix [28]. Under these conditions, the value of 0.90 [25] can be adopted for the exponent q.

For these considerations, from Relation (5), the force multiplication coefficient determined by simulation with DEM, *F_z_*_1_, is extracted, having the value

(*D*_1_/*D*_2_)*^q^* = (1140/140)^0.9^ = 6.602 (6)

This value should be used to multiply the values of the horizontal component of the milling force, *F_z_*_1_, in order to make a relevant comparison with the values of the force *F_z_*_2_, obtained experimentally.

Normally, the wear of the cutter teeth is manifested uniformly on the surface of the active flank (in the case of asphalt cutters, in a direction perpendicular to the generator of the active cone), influencing, directly proportionally, the value of the cutting forces. The component of the resultant cutting forces in the forward direction of the cutting tool (horizontal component) is determined with Formula [29]:

*F_H_* = μ · *P_H_* · *W_f_* · *B* (7)

where μ is the coefficient of friction between the active surface of the tooth and the processed material; *P_H_* is the average specific pressure on the active surface of the tooth; *W_f_* is the amount of tooth wear; and *B* is the length of the cutting edge in contact with the chipped material.

Starting from Equation (7), the study of the cutting forces is justified in order to establish optimal milling conditions that guarantee a minimum wear of the cutter teeth. Therefore, the experimental research was directed towards identifying the influence of the cutting parameters on the intensity of the cutting forces.

### 2.4. Conditions for Running the Experimental Program

The procedure for carrying out the experimental tests assumed the establishment of operating conditions (milling) in correlation with the operating possibilities of the machine tool, on the basis of which the experimental device was designed. In this sense, the first experimental program was based on the use of 30 mm milling depth, performing milling operations using a range of six distinct values for advance speed: 30, 75, 118, 190, 235, 375 mm/min, and three values for the rotation speed: 30, 75, 300 rpm. The experimental results obtained—in the form of the horizontal component of the cutting force (milling)—were compared with the results obtained from the DEM modeling of the milling process, under the conditions of using the same working parameters as during the experiment. The purpose of this comparison was to establish the extent to which DEM modeling can be used to estimate the forces developed in milling. A second experimental program used a single rotation speed—75 rpm—changing the values of the milling depth—15, 30, 50 mm—and the advanced speed—190, 235, 375 mm/min—in order to establish the contribution of each of the two parameters on the value of the horizontal force during milling operation.

The choice of parameters for the second experimental program was based on the intention to bring the experimental conditions as close as possible to the actual operating conditions of asphalt milling machines. In real conditions, asphalt milling machines can have working parameter values as follows [30]:

- drum speed, between 0–210 rpm;
- advance speed, between 0–15 (30) m/min.

In these conditions, taking into account the possibilities of the experimental stand, the pairs of values stated above were chosen.

In our experiments, the values of rotation speed were 30, 75, 300 rpm (the value of 300 rpm was chosen to analyze the effect of an extreme value of rotation on the cutting forces). Also, the values of advance speed were chosen at the inferior limit of the real advance speed presented above.

### 2.5. DOE Analysis

As already was discussed, the cutting resistance, which is a crucial design factor, is influenced by various factors such as milling depth, rotation speed, advance speed, etc. Conducting a one-factor-at-a-time analysis would necessitate an impractically large number of DEM simulations or experiments due to the significant variations under different work conditions. To address this challenge, statistical methods for Design of Experiments (DOE) offer a more efficient approach [31]. Design of Experiments (DOE) is a systematic approach used to plan, conduct, and analyze experiments in order to understand and optimize processes or systems. By varying input factors systematically and observing their effects on output responses, DOE helps to uncover relationships between variables, identify optimal conditions, and minimize variability. One of the primary advantages of DOE is its efficiency in experimentation. Rather than relying on ad hoc or one-factor-at-a-time approaches, DOE enables researchers to evaluate multiple factors simultaneously, often with fewer experiments. This efficiency is particularly valuable in situations where conducting experiments is time-consuming, expensive, or resource intensive.

Another advantage of DOE is its ability to identify important factors and interactions that may not be apparent when examining variables individually. Through statistical analysis techniques such as analysis of variance (ANOVA) or regression analysis, DOE helps distinguish between significant and insignificant factors, as well as quantify the effects of different factors and their interactions on the response variables. Furthermore, DOE facilitates optimization by guiding researchers towards optimal process conditions or parameter settings. By systematically exploring the design space and identifying optimal factor settings that maximize or minimize desired responses, DOE helps to improve process efficiency, product quality, and overall performance.

In this study, we utilized the full factorial design method with Minitab 19 software to examine the impact of milling parameters, namely, milling depth and advance speed, on cutting forces. Two input parameters were introduced with three levels each, as outlined in Table 4, and the experimental and numerical results are presented in Table 5 and Table 6.

## 3. Results and Discussion

### 3.1. Comparative Analysis Regarding the Influence of Cutting Parameters on the Resistance Force during Asphalt Milling

**Figure 8:**
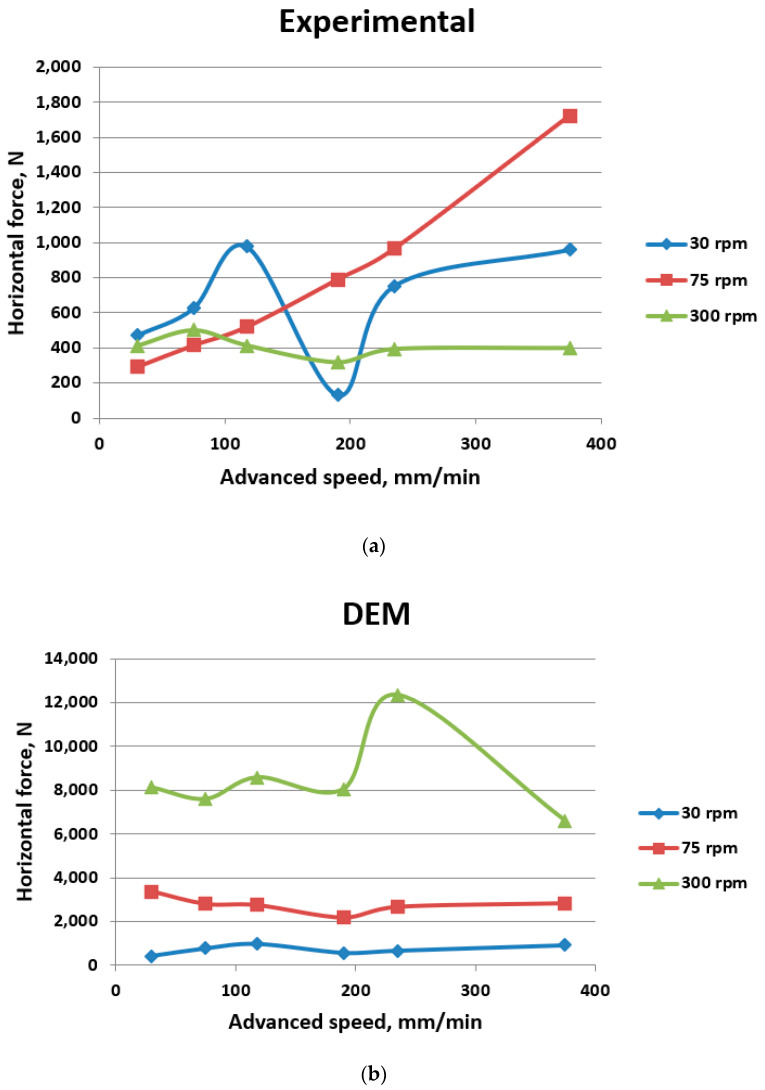
The influence of advanced speed and rotation speed on horizontal cutting for 30 mm milling depth: (**a**) experimental results; (**b**) numerical results.

The first investigation was performed considering the same milling depth *a_p_* = 30 mm and different values of rotation speed and advanced speed. The results of horizontal cutting force, obtained experimentally and numerically, are presented in Table 5 and Figure 8.

The numerical results show that, as the rotation speed increases, the horizontal cutting force generally tends to increase. Conversely, as the advanced speed increases, the horizontal cutting force typically increases.

There is variability in the results between experimental and numerical (DEM) values, with some instances showing close agreement and others showing significant discrepancies. These differences can be attributed to the simplification made in numerical analysis, where spherical particles were used in order to reduce the computational time.

Generally, the horizontal cutting force values from numerical simulations (DEM) are notably higher than the experimental values.

### 3.2. Statistical Response Analysis Based on Numerical and Experimental Results of the Cutting Force

#### 3.2.1. The Results of Numerical and Experimental Investigation

**Figure 9:**
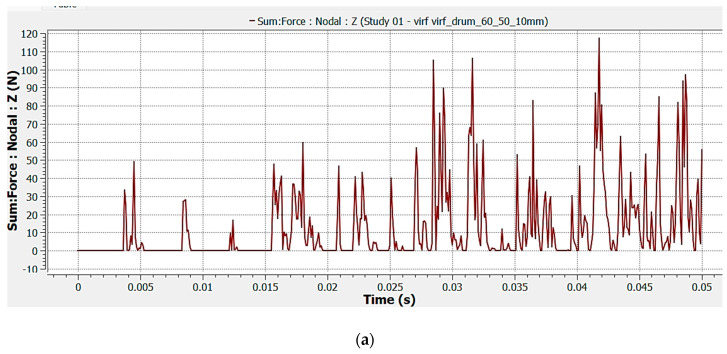

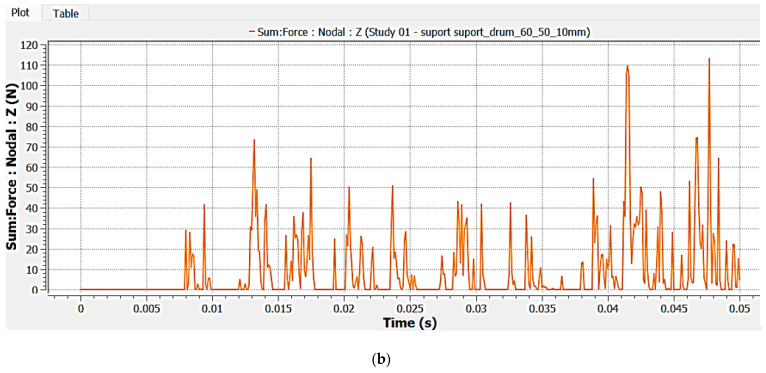
Horizontal cutting force variation during milling process simulation for (**a**) tooth tip; (**b**) tooth support.

In DEM analysis, the values of cutting forces were determined in the *Oz* direction (the horizontal direction that coincides with the advance direction), both for tooth support and tip (one example is presented in Figure 9), and the total force was determined by summing them. The cutting forces were established by identifying the peak values obtained from summing the forces during the simulation of the milling process.

**Figure 10:**
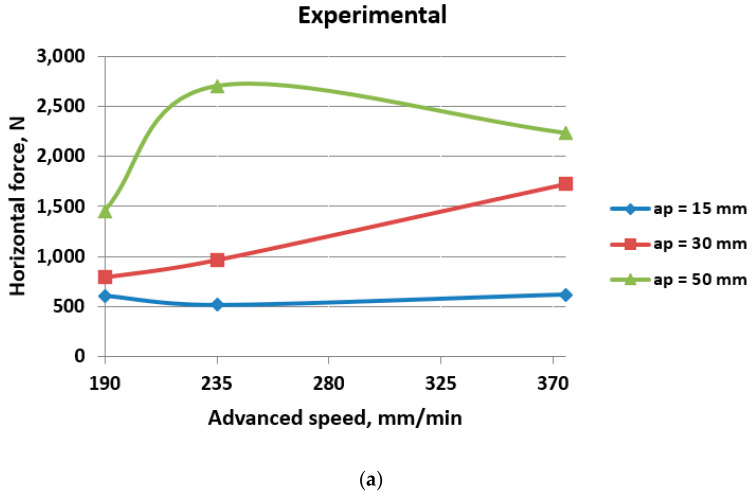

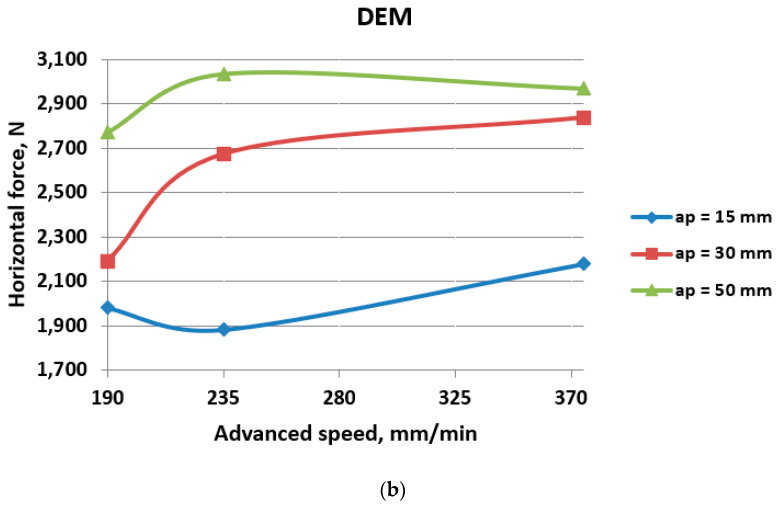
Horizontal cutting force as a function of advanced speed: (**a**) experimental results; (**b**) numerical results.

**Figure 11:**
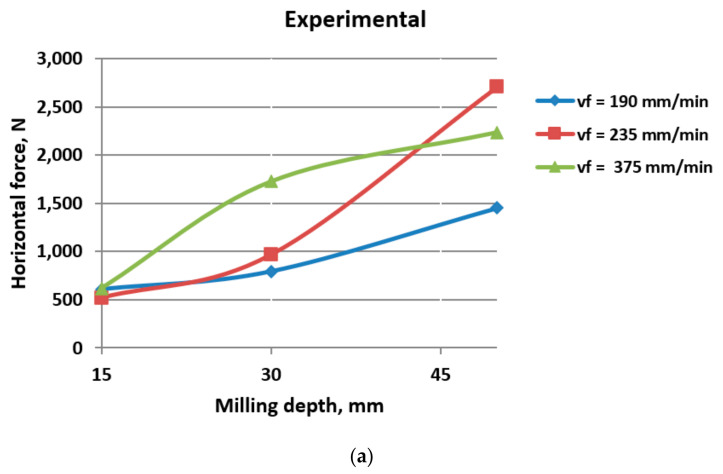

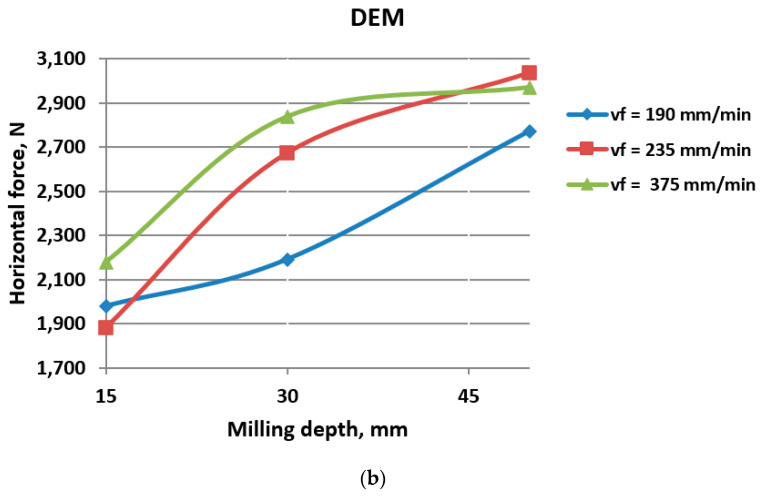
Horizontal cutting force as a function of milling depth: (**a**) experimental results; (**b**) numerical results.

The comparative results regarding the values of horizontal cutting forces can be seen in Table 6 and Figure 10 and Figure 11.

As the milling depth increases from 15 mm to 50 mm, the horizontal cutting force generally tends to increase. This trend suggests that deeper milling operations require higher cutting forces to achieve the desired material removal.

Within each milling depth, there is variation in the horizontal cutting force with different advanced speeds. Generally, higher advanced speeds correspond to higher cutting forces, indicating that faster feed rates require greater force to maintain the milling process. In one previous work [3], DEM results showed that, for small milling depth values, the advanced speed does not significantly influence the cutting forces. However, for large milling depths, specifically, 100 mm and higher, there is a substantial increase in force values.

In most cases, the numerical results are notably higher than the experimental values. This difference might arise from various factors such as simplifications in the numerical model or inaccuracies in material properties. These discrepancies underscore the challenges in accurately modeling and simulating milling processes. In any case, despite the variance between the experimental and numerical values of cutting force, the graphs exhibit a consistent pattern regarding the impact of milling parameters. This consistency suggests that, although there may be discrepancies in the absolute values obtained through experimental and numerical methods, the relative influence of milling parameters on cutting force remains similar across both approaches.

This alignment underscores the robustness of the observed trends and reinforces the significance of milling parameters, such as depth and advanced speed on horizontal cutting force. Such convergence between experimental and numerical results enhances confidence in the validity of the findings and lends further credence to the importance of optimizing milling parameters for minimizing cutting force in machining processes.

Overall, the data highlight the influence of milling depth and advanced speed on the horizontal cutting force during milling operations.

#### 3.2.2. Statistical Analysis Regarding the Influence of Milling Parameters on the Cutting Forces Acting on the Tooth

**Figure 12:**
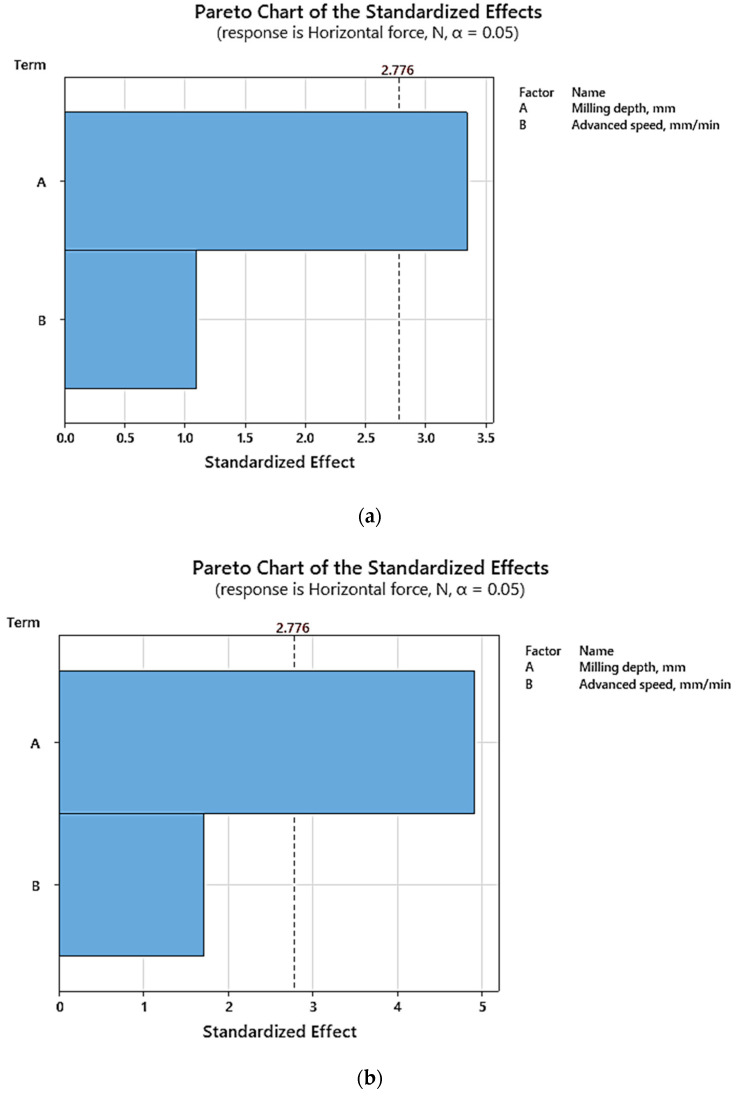
Pareto charts based on (**a**) experimental results; (**b**) numerical results.

**Figure 13:**
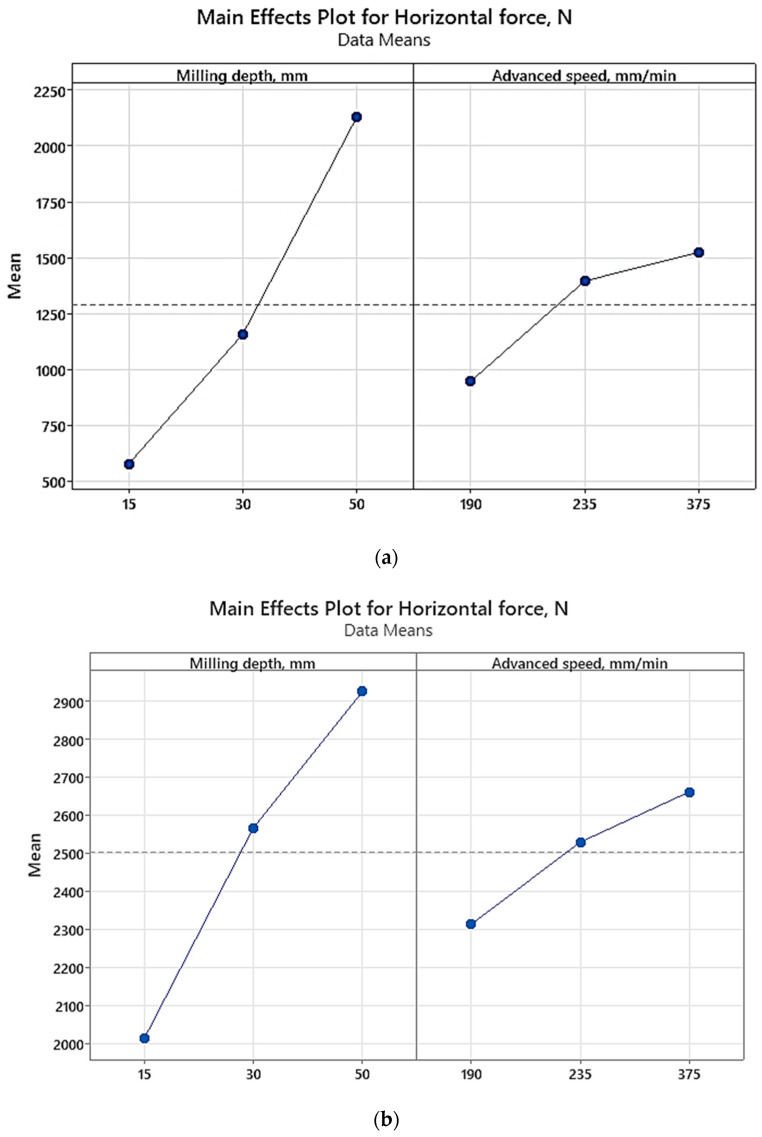
Main effects plots based on (**a**) experimental results; (**b**) numerical results.

The graphs in Figure 12 and Figure 13 highlight that milling depth emerges as the primary factor influencing cutting force. This observation resonates with previous studies [3,32,33], which underscored the pivotal role of cutting depth in determining resultant force.

Moreover, the graphical representations in Figure 13 facilitate the identification of the optimal milling parameter combination for minimizing cutting force. Specifically, they suggest that a milling depth of 15 mm paired with an advanced speed of 190 mm/min constitutes the most favorable configuration for achieving this objective.

#### 3.2.3. Regression Analysis

A. Constant milling depth

Based on the experimental obtained results, the expression of resistance force *F_z_* was obtained as a function of advanced speed *v_f_* as

(8) $$F_{z}=f(v_{f})=339.421+1.618\cdot v_{f}$$

Above this dependence, the mediating factor rotation speed *n* is introduced. Its influence, as can be seen from the mathematical model, is not significant:

(9) $$F_{z}=f(v_{f},n)=496.590-1.164\cdot n+1.618\cdot v_{f}$$

For significance coefficients α*_n_* = 0.074, α*_vf_* = 0.021, and *R* = 0.638, *R*^2^ = 0.407.

The significance coefficients suggest that the advanced speed *v_f_* has a more significant impact on the resistance force compared to the rotation speed *n*. Additionally, the coefficient of determination (*R*^2^ = 0.407) indicates that approximately 40.7% of the variability in the resistance force can be explained by the advanced speed alone. While the coefficient of determination is moderate, it suggests that there are other factors not captured by the model that contribute to the variability in the resistance force.

B. Constant rotation speed

Based on the experimental obtained results, the expression of resistance force *F_z_* was obtained as function of milling depth *a_p_* as

(10) $$F_{z}=f(a_{p})=-120.396+44.542\cdot a_{p}$$

Above this dependence, the mediating factor advanced speed *v_f_* is introduced:

(11) $$F_{z}=f(v_{f},a_{p})=-810.427+44.543\cdot a_{p}+2.588\cdot v_{f}$$

**Figure 14:**
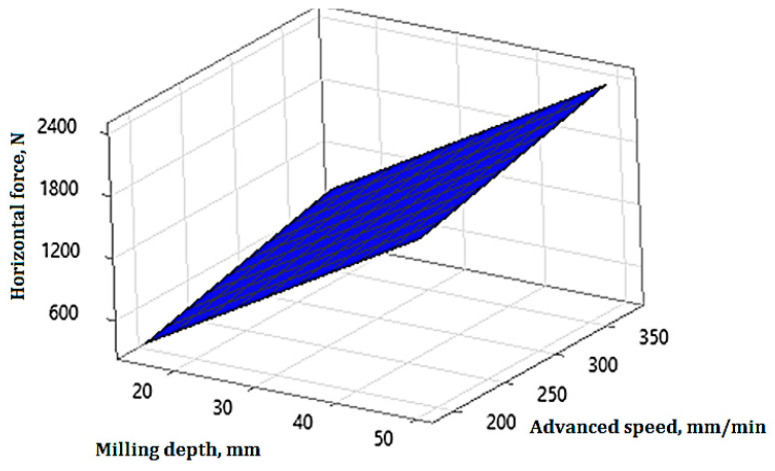
The 3D surface plot representation for horizontal force.

The surface plot described by Equation (11) is shown in Figure 14.

Table 7 presents the ANOVA coefficients for regression Equation (11).

The constant term in the regression equation (in this case, −810) does not seem to be statistically significant, as its *p*-value is 0.191 (>0.05).

The coefficient milling depth variable is 44.54, meaning that, for every 1 mm increase in milling depth, the dependent variable (horizontal force) increases by 44.54 units on average. This coefficient is statistically significant with a *p*-value of 0.003 (<0.05), indicating that milling depth has a significant impact.

The coefficient advanced speed for this variable is 2.59. This means that, for every 1 unit increase in advanced speed (measured in mm/min), the dependent variable (horizontal force) increases by 2.59 units on average. However, the *p*-value associated with this coefficient is 0.173 (>0.05), indicating that this effect is not statistically significant at the significance level of 0.05.

VIF (Variance Inflation Factor): VIF measures the extent of multicollinearity in the regression model. Since all VIF values are 1.00, this indicates no issues with multicollinearity.

In summary, milling depth seems to have a significant impact on the dependent variable, while advanced speed does not appear to have a statistically significant effect.

*R* = 0.901; *R*^2^ = 0.811.

The significance coefficients have acceptable, slightly high values. This can be attributed to the inhomogeneity of the processed material (asphalt), which introduces a certain degree of uncertainty in modeling. Despite this uncertainty, the regression models provide valuable insights into the relationships between the variables studied, offering a basis for further exploration and refinement in understanding asphalt milling processes. The coefficient of determination (*R*^2^) measures the proportion of variability in the resistance force (*F_z_*) that can be explained by the combined effects of milling depth and advanced speed. In this case, *R*^2^ = 0.811, indicating that approximately 81.1% of the variability in the resistance force can be explained by the regression model that contains milling depth and advanced speed as predictors. This suggests that the model fits the data well and provides a good representation of the relationship between the independent variables and the dependent variable.

**Figure 15:**
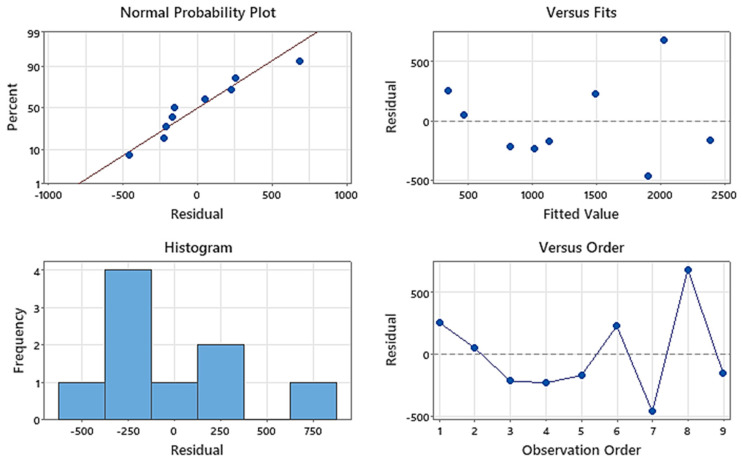
Residual plots for horizontal force.

The residual plots from Figure 15 suggest that the regression assumptions are generally satisfied: residuals are approximately normally distributed, show constant variance and no apparent autocorrelation in residuals appears.

In order to analyze statistically if there are interactions between the input variables, we chose a regression model that included the interaction term $a_{p}\cdot v_{f}$, resulting in the regression equation for resistance force *F_z_* and the ANOVA results in Table 8.

(12) $$F_{z}=f(v_{f},a_{p})=-369+30.6\cdot a_{p}+0.93\cdot v_{f}+0.052\cdot a_{p}\cdot v_{f}$$

The data from Table 8 show that the interaction term (Milling depth · Advanced speed) has a *p*-value of 0.695, which is significantly greater than the common level of 0.05. This indicates that there is no significant interaction between the input variables. The contribution of 0.63% indicates that the interaction term between milling depth and advanced speed explains a very small part of the total variation in the response variable. This minimal contribution, along with the high *p*-value (0.695), justifies the exclusion of the interaction term from the model, leading to a simpler and more interpretable model focusing on the significant main effects.

## 4. Conclusions

In this study, the authors designed and used a specialized experimental stand to investigate the milling process of asphalt samples in laboratory conditions. The stand, as described in detail in Section 2.1, allowed for controlled variation of various parameters affecting the milling process, including milling depth, rotational speed, and advanced speed. Moreover, for the experiments, the authors utilized a specific cutter tooth design (Wirtgen type) made of steel with a tungsten carbide tip. This tooth design is commonly employed in asphalt milling applications due to its durability and effectiveness. By focusing on the interaction between a single cutting element of the milling drum and the asphalt sample, we aimed to determine the forces involved in the cutting process and their relationship to the geometric and dynamic parameters. The main conclusions of the performed investigation are as follows:

✓ The numerical results indicate that an increase in rotational speed generally leads to an increase in horizontal cutting force. Conversely, an increase in advance speed typically results in a decrease in horizontal cutting force;
✓ Within each milling depth, the horizontal cutting force varies with different advance speeds. Generally, higher advance speeds correspond to higher cutting forces, indicating that faster feed rates require greater force to sustain the milling process;
✓ Milling depth emerges as the primary factor influencing cutting force, highlighting its critical role in the milling process;
✓ Through experimental, numerical, and statistical analysis, it was determined that the optimal combination of milling parameters to achieve minimum horizontal force acting on the cutting tooth are 15 mm milling depth and 190 mm/min;
✓ Based on the optimization results, new, economically viable operation modes of equipment, along with rational design and technological parameters of machines, can be identified. The identified optimal milling parameters not only contribute to reducing cutting forces but also have implications for minimizing tooth wear. By minimizing the forces exerted on the cutting tooth, this results in reduced mechanical stress and friction experienced by the cutting tooth, leading to less abrasive wear and fatigue, resulting in extended tool life. A reduction in tooth wear means fewer instances of tool replacements, leading to cost savings and improved operational efficiency;
✓ Despite discrepancies between experimental and numerical results, consistent trends in the relative influence of milling parameters on cutting force were observed, emphasizing the importance of optimizing parameters for reducing cutting force in machining processes;
✓ However, it is important to specify that not all process parameters can be replicated on such machines in the same way as they operate on real machines. Cutting speeds on such equipment will notably be lower, potentially resulting in reduced cutting resistance forces. Additionally, refining DEM models to better replicate the inhomogeneity and anisotropy of real asphalt materials, exploring the application of the identified optimal milling parameters in actual road construction and maintenance scenarios to validate the laboratory findings and developing new cutter tooth designs and materials to further enhance tool durability and performance in asphalt milling applications remain topics for further investigations;
✓ The scientific contribution of this research includes the development of an original experimental program to determine the forces involved in asphalt milling and the validation of numerical modeling (DEM) as a cost-effective alternative to physical experiments. This study enhances the understanding of the relationship between milling parameters and cutting forces, providing valuable data for optimizing milling operations;
✓ The experimental results can offer solutions for choosing the milling parameters to minimize the values of cutting forces. Reducing the forces will result in reducing the cutting tool wear [2] and increasing its durability;
✓ Overall, the experimental setup and methodology described herein provide a base for studying the asphalt milling process and understanding the factors influencing cutting forces and material removal. The insights gained from this study have implications for optimizing milling operations in road construction and maintenance, potentially leading to improvements in efficiency and performance.

## Figures and Tables

**Table 1:** Mechanical and dimensional characteristics of milling machine FU 32 × 132.

Name of Characteristic	Characteristic Value
Table length, mm	1325
Table width, mm	320
Table working area, mm	320 × 1325
Length of table travel on axis: X/Y/Z, mm	700/250/370
Table turning angle, degrees	+/−45
Number of main spindle speed steps	18
Main spindle speed steps limits, rot/min	30–1500
Number of working advance/feed steps	18
Limits of working advance steps, mm/min: longitudinal, transversal/vertical	19–950/¼ of the mentioned values
Fast table feed, mm/min: longitudinal, transversal/vertical	2300/ ¼ of the mentioned value
Main electric motor power, kW	7.5
Secondary electric motor power (for feed movements), kW	2.2
Net mass, kg	3100

**Table 2:** Composition of asphalt concrete.

Material		Composition, %
Mineral aggregate	Size 4–8 mm	22
	Size 8–16 mm	43
Sand		19
Filler		10
Bitumen		6

**Table 3:** The working parameters used during the two stages of the experimental program.

Milling Depth, *a_p_* [mm]	Milling Drum Rotation Speed, *n* [rpm]	Advanced Speed, *v_f_* [mm/min]	Angle of Attack, α [°]
15/30/50	30/75/300	30/75/118/190/235/375	60

**Table 4:** Parameters and levels used in DOE analysis.

Test No.	Parameters	
	Milling Depth, mm	Advanced Speed, mm/min
1	15	190
2	15	235
3	15	375
4	30	190
5	30	235
6	30	375
7	50	190
8	50	235
9	50	375

**Table 5:** Experimental and numerical values of horizontal cutting force for 30 mm milling depth.

Crt. No.	Rotation Speed, rpm	Advanced Speed v*f*, [mm/min]	Horizontal Cutting Force *F_z_*, [N]	
			Experimental, *F_z_*_1_	DEM (Multiplied with *q* According to (5), *F_z_*_2_)
1.	30	30	472	409
2.		75	627	775
3.		118	978	990
4.		190	133	555
5.		235	754	660
6.		375	962	924
7.	75	30	292	3367
8.		75	415	2806
9.		118	522	2751
10.		190	791	2191.2
11.		235	965	2673
12.		375	1724	2838
13.	300	30	412	8118
14.		75	502	7590
15.		118	414	8580
16.		190	317	8052
17.		235	394	12,342
18.		375	400	6600

**Table 6:** Experimental and numerical values of horizontal cutting forces for 75 rpm drum rotation speed and different cutting parameters.

Milling Depth, mm	Advanced Speed, mm/min	Horizontal Force, N		
		Experimental	DEM	DEM Multiplied by *q*
15	190	605	300	1980
15	235	518	285	1881
15	375	616	330	2178
30	190	791	332	2191.2
30	235	965	405	2673
30	375	1724	430	2838
50	190	1452	420	2772
50	235	2706	460	3036
50	375	2234	450	2970

**Table 7:** ANOVA coefficients.

Term	Coef	SE Coef	T-Value	*p*-Value	VIF
Constant	−810	550	−1.47	0.191	
Milling depth, mm	44.54	9.20	4.84	0.003	1.00
Advanced speed, mm/min	2.59	1.68	1.54	0.173	1.00

**Table 8:** ANOVA results.

Source	DF	Seq SS	Contribution	Adj SS	Adj MS
Regression	3	4,075,761	81.76%	4,075,761	1,358,587
Milling depth, mm	1	3,670,437	73.63%	139,069	139,069
Advanced speed, mm/min	1	373,958	7.50%	8260	8260
Milling depth, mm·Advanced speed, mm/min	1	31,365	0.63%	31,365	31,365
Error	5	909,142	18.24%	909,142	181,828
Total	8	4,984,903	100.00%		
Source	F-Value			*p*-Value	
Regression	7.47			0.027	
Milling depth, mm	0.76			0.422	
Advanced speed, mm/min	0.05			0.840	
Milling depth, mm·Advanced speed, mm/min	0.17			0.695	

## Data Availability

The original contributions presented in the study are included in the article, further inquiries can be directed to the corresponding authors.

## References

[1] Dumitru T., Ilincă C., Tănase M. (2022). Influence of Technological Parameters on the Behaviour in Operation of the Asphalt Milling Equipment. IOP Conf. Ser. Mater. Sci. Eng..

[2] Dumitru T., Petrescu M.G., Tănase M., Laudacescu E. (2023). The Application of Tribological Tests to Study the Wear Behavior of Asphalt Cutter Teeth: An Experimental Investigation Using Baroid Tribometer. Coatings.

[3] Dumitru T., Petrescu M.G., Tănase M., Ilincă C.N. (2023). Multi-Response Optimization Analysis of the Milling Process of Asphalt Layer Based on the Numerical Evaluation of Cutting Regime Parameters. Processes.

[4] Furmanov D.V., Lysakov N.E., Shamakhov L.M. (2022). Experimental and Analytical Justification of the Asphalt Concrete Cutting Process by Road Milling Machines Working Equipment. Russ. Automob. Highw. Ind. J..

[5] Furmanov D.V., Shamakhov L.M., Lysakov N.E. (2023). Wear out Effect for Cutting Element of Milling Machine on Asphalt Concrete Cutting Strength. Russ. Automob. Highw. Ind. J..

[6] Furmanov D.V. (2021). Comparative Analysis of Experimental Methods to Assess the Resistance of Milling Road Asphalt Concrete. IOP Conf. Ser. Mater. Sci. Eng..

[7] Bobrenkov O.A., Khasawneh F.A., Butcher E.A., Mann B.P. (2010). Analysis of Milling Dynamics for Simultaneously Engaged Cutting Teeth. J. Sound Vib..

[8] Wu J., Li D., Zhu B., Wu C. (2018). Milling Process Simulation of Old Asphalt Mixture by Discrete Element. Constr. Build. Mater..

[9] Varga J., Demko M., Kaščák Ľ., Ižol P., Vrabeľ M., Brindza J. (2024). Influence of Tool Inclination and Effective Cutting Speed on Roughness Parameters of Machined Shaped Surfaces. Machines.

[10] Zagórski I., Zgórniak P., Habrat W., Machado J., Legutko S. (2024). Methodology of Chip Temperature Measurement and Safety Machining Assessment in Dry Rough Milling of Magnesium Alloys Using Different Helix Angle Tools. Materials.

[11] Zawada-Michałowska M., Pieśko P., Mrówka-Nowotnik G., Nowotnik A., Legutko S. (2024). Effect of the Technological Parameters of Milling on Residual Stress in the Surface Layer of Thin-Walled Plates. Materials.

[12] Sebbe N.P.V., Fernandes F., Silva F.J.G., Pedroso A.F.V., Sales-Contini R.C.M., Barbosa M.L.S., Durão L.M., Magalhães L.L. (2024). Wear Behavior of TiAlVN-Coated Tools in Milling Operations of INCONEL^®^ 718. Coatings.

[13] Kowalczyk M. (2024). Analysis of Cutting Forces and Geometric Surface Structures in the Milling of NiTi Alloy. Materials.

[14] Jiang B., Nie Q., Zhao P., Ma Q., Sun S. (2023). Identification Method for Instantaneous Friction and Wear Energy Density Variation of High-Feed Milling Tool Flank. Appl. Sci..

[15] Sivilevičius H., Martišius M. (2022). Field Investigation and Assessment on the Wear of Asphalt Pavement Milling Machine Picks. Transport.

[16] Furmanov D.V., Lysakov N.E., Shamahov L.M., Klyuev S.V., Klyuev A.V., Vatin N.I. (2021). Experimental Justification of Geometrical Model of Cut Chip Cross-Section during Asphalt Milling. Innovations and Technologies in Construction.

[17] Pirnaev S., Sindarov R., Dzhumabeva F., Saidova S. (2021). Technique for Experimental Studies of Asphalt Concrete Milling Process. E3S Web Conf..

[18] Furmanov D., Chizhov V., Tyuremnov I., Troshin D. (2019). Loads on Cutter Teeth for Removing Asphalt Pavement. E3S Web Conf..

[19] Blum J., Anderegg R. (2016). Modelling of an Innovative Technology for Pavement Milling. IFAC-Pap..

[20] Guan Y., Guan H. (2019). Algorithms for Modelling 3D Flexible Pavements and Simulation of Vibration Cutting by the DEM. Int. J. Pavement Eng..

[21] Diouri K., Bousselham R., De A., Hera A., El-Korchi T., Mallick R.B., Raab C. (2020). A Study on the Effect of Milling on Stress Distributions in Asphalt Pavements. Proceedings of the 9th International Conference on Maintenance and Rehabilitation of Pavements—Mairepav9.

[22] Nooraie R.Y., Safari M., Pak A. (2020). Tool Wear Estimation in Machining Based on the Flank Wear Inclination Angle Changes Using the FE Method. Mach. Sci. Technol..

[23] Zhou L., Liu Y., Wang Z., Li Y., Zhang K., Zhang G. Numerical Analysis of Asphalt Concrete Milling Process Based on Multicomponent Modeling. Review.

[24] Makange N.R., Ji C., Torotwa I. (2020). Prediction of Cutting Forces and Soil Behavior with Discrete Element Simulation. Comput. Electron. Agric..

[25] Picos C. (1992). Proiectarea Tehnologiilor de Prelucrare Mecanică Prin Aşchiere.

[26] Hardness—Mohs Hardness Scale Asphalt Transparent PNG—600 × 600—Free Download on NicePNG. https://www.nicepng.com/ourpic/u2r5w7o0i1r5a9r5_hardness-mohs-hardness-scale-asphalt/.

[27] Hard Aggregate Source Location Study. https://dot.alaska.gov/stwddes/desmaterials/assets/pdf/hard_ashpalt_aggregate_study/final_report_hard_aggregate_study_4.pdf.

[28] What Is the Mohs Hardness for Cast Iron?. Google Will Only Show Me the Hardness for Iron, Which Is 4, but I Presume Cast Iron Will Differ Due to the Carbon and What Not.—Quora..

[29] Cutting Force Model for Tool Wear Estimation. https://websites.umich.edu/~ykoren/uploads/Cutting_Force_Model_for_Tool_Wear_Estimation.pdf.

[30] WIRTGEN Products|Wirtgen. https://www.wirtgen-group.com/ocs/en-us/wirtgen/wirtgen-products-95-c/.

[31] Jiang Y.-Z., Liao G.-W., Zhu S.-S., Hu Y.-F. (2021). Investigation on Cutting Resistance Characteristic of Bucket Wheel Excavator Using DEM and DOE Methods. Simul. Model. Pract. Theory.

[32] Kanchana J., Prasath V., Krishnaraj V., Geetha Priyadharshini B. (2019). Multi Response Optimization of Process Parameters Using Grey Relational Analysis for Milling of Hardened Custom 465 Steel. Procedia Manuf..

[33] Kalyon A., Günay M., Özyürek D. (2018). Application of Grey Relational Analysis Based on Taguchi Method for Optimizing Machining Parameters in Hard Turning of High Chrome Cast Iron. Adv. Manuf..

